# Thromboxane–prostaglandin receptor antagonist, terutroban, prevents neurovascular events after subarachnoid haemorrhage: a nanoSPECT study in rats

**DOI:** 10.1186/s13054-019-2338-4

**Published:** 2019-02-11

**Authors:** David Lagier, David Tonon, Philippe Garrigue, Benjamin Guillet, Laura Giacomino, Jean-Charles Martin, Marie-Christine Alessi, Nicolas Bruder, Lionel J. Velly

**Affiliations:** 1 Department of Anaesthesiology and Critical Care Medicine, University Hospital Timone, Marseille, France; 2 C2VN Inserm 1263, Inra 1260, Aix Marseille University, Marseille, France; 3 CERIMED (European Center for Research in Medical Imaging), Aix Marseille University, Marseille, France; 4 Department of Anaesthesiology and Critical Care Medicine, INT (Institut de Neurosciences de la Timone), University Hospital Timone, Aix Marseille University, Marseille, France

**Keywords:** Subarachnoid haemorrhage, Single-photon emission computerized tomography, Thromboxane–prostaglandin receptor, Cerebral vasospasm, Blood brain barrier, Apoptosis

## Abstract

**Background:**

Several lipid metabolites in cerebrospinal fluid are correlated with poor outcomes in aneurysmal subarachnoid haemorrhage. Most of these metabolites bind to ubiquitous thromboxane–prostaglandin (TP) receptors, causing vasoconstriction and inflammation. Here, we evaluated terutroban (TBN), a specific TP receptor antagonist, for the prevention of post-haemorrhage blood-brain barrier disruption, neuronal apoptosis and delayed cerebral hypoperfusion.

**Methods:**

The rat double subarachnoid haemorrhage model was produced by twice injecting (days 1 and 2) autologous blood into the cisterna magna. Seventy-eight male Sprague-Dawley rats were assigned to experimental groups. Rats exposed to subarachnoid haemorrhage were allocated to no treatment (SAH group) or TBN treatment by gastric gavage during the first 5 days after haemorrhage (SAH+TBN group). Control rats received artificial cerebrospinal fluid injections (CSF group). Sham-operated rats with or without TBN administration were also studied. Body weight and Garcia neurological scores were assessed on day 2 and day 5. We used nanoscale single-photon emission computed tomography (nanoSPECT) to measure brain uptake of three radiolabelled agents: ^99m^Technetium-diethylenetriaminepentacetate (^99m^Tc-DTPA), which indicated blood-brain barrier permeability on day 3, ^99m^Technetium-annexin V-128 (^99m^Tc-Anx-V128), which indicated apoptosis on day 4, and ^99m^Technetium-hexamethylpropyleneamineoxime (^99m^Tc-HMPAO), which indicated cerebral perfusion on day 5. Basilar artery narrowing was verified histologically, and cerebral TP receptor agonists were quantified.

**Results:**

^99m^Tc-DTPA uptake unveiled blood-brain barrier disruption in the SAH group. TBN mitigated this disruption in the brainstem area. ^99m^Tc-Anx-V128 uptake was increased in the SAH group and TBN diminished this effect in the cerebellum. ^99m^Tc-HMPAO uptake revealed a global decreased perfusion on day 5 in the SAH group that was significantly counteracted by TBN. TBN also mitigated basilar artery vasoconstriction, neurological deficits (on day 2), body weight loss (on day 5) and cerebral production of vasoconstrictors such as Thromboxane B2 and Prostaglandin F2α.

**Conclusions:**

Based on in vivo nanoscale imaging, we demonstrated that TBN protected against blood-brain barrier disruption, exerted an anti-apoptotic effect and improved cerebral perfusion. Thus, TP receptor antagonists showed promising results in treating post-haemorrhage neurovascular events.

## Background

Most survivors of a cerebral aneurysm rupture experience subarachnoid haemorrhage (SAH)-related morbidity and mortality, due to early brain injury and delayed cerebral ischemia [1]. Recent studies have highlighted a complex pathophysiology of post-SAH neurovascular events [2] that extends beyond cerebral vasospasm [3]. First, early brain injury implies microvascular spasms [4], loss of arteriolar autoregulation [5], apoptosis [6] and microthrombosis [7]. Then, inflammatory response and oxidative stress induced by late degradation of blood products in the subarachnoid space [8] contribute to delayed cerebral ischemia.

Arachidonic acid is metabolized through enzymatic (eicosanoid family: thromboxane A2 and prostaglandins) or non-enzymatic (F2-isoprostanes, 20-hydroxyeicosatetraeonic acid) pathways. Modification of arachidonic acid metabolism has been identified in a rat model of SAH [9]. Then, the CSF level of these metabolites was positively associated with the development of cerebral vasospasm and the neurological outcome in humans [10–13]. These lipid metabolites specifically bind the thromboxane and prostaglandin (TP) receptors [14]. TP receptor activation leads to platelet or endothelial cell activation, smooth muscle cell contraction [15], endothelin-1 production and leucocyte recruitment [16]. At the vascular level, these agonists have shown [17] to promote vasospasm, thrombosis and parietal remodelling [14, 18, 19]. Similarly, the only effective and currently available treatment, nimodipine, reduced the production of thromboxane–prostaglandin (TP) receptor agonists in a rabbit model of SAH [20]. These data suggest that TP receptor antagonists represent interesting tools to prevent brain damage following SAH.

Terutroban (TBN), a specific TP receptor antagonist [21], was first studied as an antiplatelet and vasoprotective agent. Lesault et al. [22] revealed that TBN improved endothelial function and arterial vasodilation properties in an atherosclerotic population. However, in a large clinical trial with mid-term outcomes, TBN failed to show clinical relevance compared to aspirin for the secondary prevention of cardiovascular events in stroke patients [23]. Subsequently to this negative result, TBN development was stopped by the industry. However, unlike atherosclerosis, SAH is associated with a massive, localized and acute production of TP receptor agonists, mediated by inflammatory and oxidative pathways [10, 12, 13, 24]. Moreover, Ansar et al. have showed, in a rat model, that intracisternal injection of blood profoundly increased neurovascular TP receptor expression [25].

The objective of this study was to evaluate the effectiveness of TBN in preventing post-SAH neurovascular events. For the in vivo study, we used functional outcomes and nanoscale single-photon emission computed tomography (nanoSPECT) to evaluate blood-brain barrier disruption, neuronal apoptosis and cerebral hypoperfusion. For the post mortem study, we evaluated vasospasm of the basilar artery and cerebral production of TP receptor agonists. The double intracisternal injection SAH rat model was used as it is validated for inducing intracranial hypertension, delayed decrease in cerebral blood flow and basilar artery vasospasm [26].

## Methods

### Animals

Adult male Sprague-Dawley rats (*n* = 78; 280–320 g; Janvier Labs, France) were housed at 21 ± 0.5 °C with food and water ad libitum on a light-dark reverse cycle. All experiments were approved by the Aix-Marseille University Ethics Committee on Animal Experimentation (CE14; n°3-17012013) and were performed in an accredited laboratory (C13-055-20).

### SAH rat model

Rat subarachnoid haemorrhage was performed as previously described by Dusick et al. [27]. Briefly, general anaesthesia was delivered with intraperitoneal injections of ketamine (100 mg/kg) and midazolam (10 mg/kg). Autologous arterial blood was collected from the ventral tail artery, and 250 μL was percutaneously injected into the cisterna magna (day 1). A stereotactic frame was used to fix the animal head by both external auditory canals and to maintain a correct anteflexion of the head. Cisterna magna was punctured percutaneously after localization of the puncture site by manual palpation of osseous landmarks. Progression of the needle was operated with a manual depression in the connected syringe. When CSF backflow appeared, the intracisternal localization of the needle tip was confirmed. Then, blood was injected. This procedure was repeated 24 h later (day 2).

### Experimental groups

**Fig. 1:**
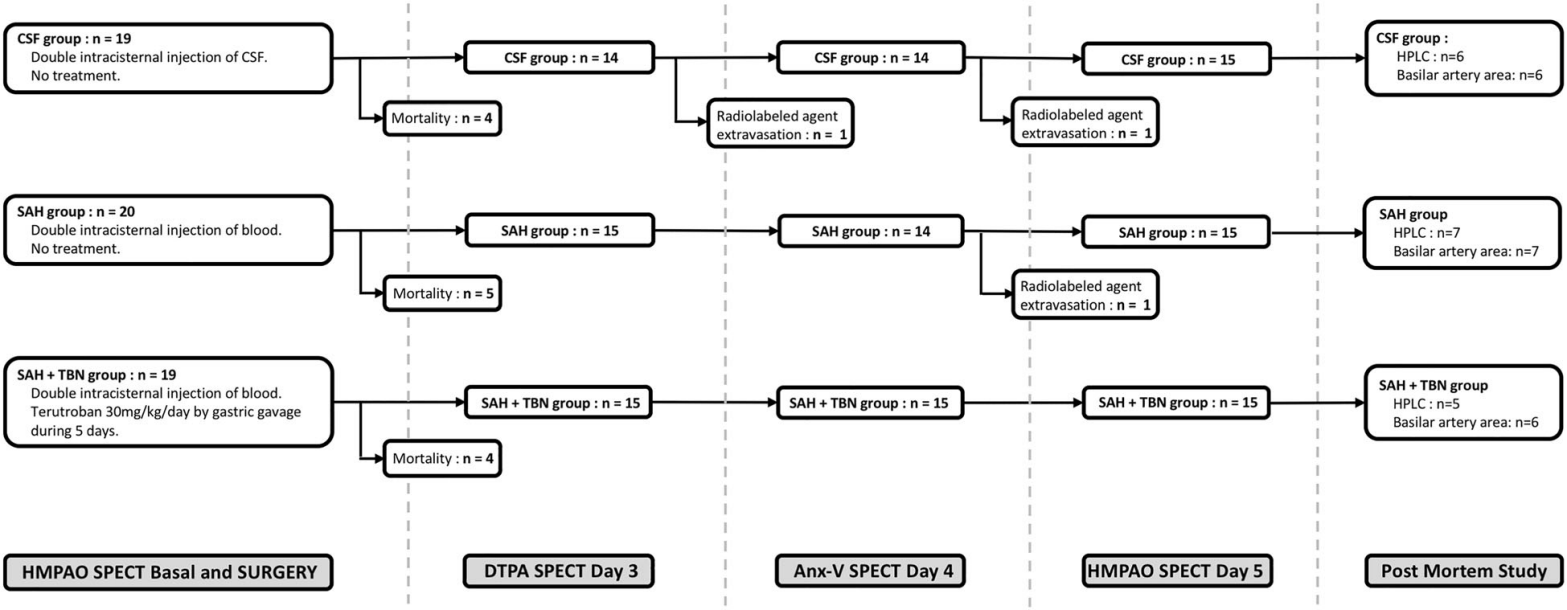
Protocol sequences. CSF, cerebrospinal fluid; HPLC, high-performance liquid chromatography; SAH, subarachnoid haemorrhage; TBN, terutroban

In the first step, rats were randomly assigned, by drawing lots, to one of the three experimental groups (Fig. 1): in the SAH group (*n* = 20), rats only received a double intracisternal injection with blood. Animals in the SAH+TBN group (*n* = 19) received a double intracisternal injection with blood and a daily TBN (Servier®, Suresnes, France) oral gavage (30 mg/kg per day dissolved in saline) from day 1 to day 5. TBN treatment was started immediately after the first intracisternal injection. The cerebrospinal fluid (CSF) group (*n* = 19) received a double intracisternal injection of 250 μL artificial CSF. Secondarily, two types of sham rats were studied: without treatment (sham group; *n* = 15) or with daily TBN oral gavage (30 mg/kg per day) from day 1 to day 5 (sham+TBN group; *n* = 5). In the sham groups, the cisterna magna was punctured without injection and any post mortem analysis was performed.

### Functional outcome

All investigators were blinded to group assignment. On days 2 and 5, body weight was recorded and rat behaviour was evaluated with the Garcia neurological scoring system [28]. The following behaviours were evaluated: spontaneous activity, symmetrical movements of limbs, forelimb outstretching, climbing the wall of a wire cage, axillary touch response, and vibrissae touch response. Each subtest was graded on a scale of 0 (worst) to 3 (best). The total score was calculated as the sum of all subtest scores.

### Evaluation of blood-brain barrier permeability, apoptosis and cerebral perfusion by NanoSPECT-CT

NanoSPECT-CT technique provides in vivo*,* longitudinal, quantitative and functional images. Imaging approaches also offer ethical advantages by largely reducing the number of animals needed (3-R rule). Blood-brain barrier permeability was studied on day 3 by injecting 20 MBq/100 μL ^99m^Tc-diethylenetriaminepentacetate (DTPA), (Pentacis®, France) into the lateral tail vein. ^99m^Tc-DTPA is a hydrophilic agent that cannot pass through an intact blood-brain barrier. ^99m^Tc-DTPA was previously used to explore blood-brain barrier disruptions in clinical studies and in other rodent models of acute neurological injuries [29]. On day 4, we evaluated the uptake of ^99m^Tc-Anx-V128 (Atreus Pharmaceuticals®, Ottawa, Canada). ^99m^Tc-Anx-V128 is a novel radiolabelled agent that target the negative phosphatidyl serine exposed on cell membranes. It is used for quantification of apoptosis. Finally, we studied the kinetics of cerebral perfusion by injecting 20 MBq/150 μL ^99m^Tc-hexamethylpropyleneamineoxime (HMPAO) (Cerestab®, GE Healthcare, France). ^99m^Tc-HMPAO, a lipophilic agent that passes through the blood-brain barrier, is metabolized in neurons, and the metabolites are trapped in the brain. This agent is well-validated for exploring cerebral perfusion in animals [30] and humans [31]. Since animals remain awake during radiotracer uptake and move freely before imaging, this technique prevents the bias of cerebral blood flow disturbances secondary to anaesthetic exposure. ^99m^Tc-HMPAO NanoSPECT images were acquired at two time points: in the basal state (day 0) and after SAH conditions were established (day 5). Forty minutes after the radiolabelled agent injection, animals were imaged with a nanoSPECT/CTplus® camera (Mediso, Hungary). Before SPECT imaging, we obtained a 0.8-mm-slice thickness CT scan of the encephalic region. During the scan, the animals were anaesthetized with isoflurane (1.5 vol%), and body temperature was maintained. Images were reconstructed and analysed with the 3D-ROI module provided in InVivoScope® software v2.0p4 (InviCRO). After co-registration between SPECT and CT scan acquisitions, three regions of interest (ROI) were drawn, based on anatomical landmarks identified in the CT images. The brainstem ROI was delimited cranially by the spheno-occipital synchondrosis and caudally by the foramen magnum. A frontal plane, passing through the fourth ventricle, delimited the cerebellum and brainstem ROIs in the antero-posterior axis. The transverse cerebral fissure was used to distinguish the cerebellum ROI from the cerebral hemispheres. Radioactivity inside each ROI was quantified and corrected according to the tissue volume (MBq/mm^3^), then divided by the total effective injected dose, after correcting for radioactive decay of ^99m^Tc. For each ROI, the radioactivity was expressed in parts per million of the injected dose (ppmID/mm^3^). The uptakes of ^99m^Tc HMPAO on day 5 were expressed as the percentage of basal uptake (day 0).

### Changes in brain TP receptor agonists

Cerebral quantification of TP receptor agonists was performed in a subgroup of 18 rats. On day 5, rats were euthanized and harvested fresh brains were frozen with liquid nitrogen. Thromboxane-B2 (TXB-2), Prostaglandins E2 (PGE2), D2 (PGD2), F2α (PGF2α) and isoprostane 8-iso Prostaglandin A-2 (8-isoPGA2) identification and quantification were performed on a half brain homogenate with high-performance liquid chromatography (HPLC; 1290 Infinity and Zorbax column SB-C18; Agilent®, Les Ulis, France) associated with tandem mass spectrometry (MS/MS; G6460 Agilent Triple Quadripole; Agilent®, Les Ulis, France). Further details are provided in [32]. Results were expressed in picograms per milligram of protein.

### Basilar artery lumen measurement

Histological examination was realized in a subgroup of 19 animals. On day 5, perfusion-fixation was performed by cannulating the left ventricle. The ventricle was perfused at 100 cmH_2_O pressure by 50 mL of 4% formaldehyde. Thereafter, the whole brain was removed and immersed in 4% formaldehyde overnight at 4 °C. Next, the brainstem was carefully dissected and embedded in paraffin. To examine the vascular changes in the basilar artery, axial brainstem slices (5 μm) were stained with haematoxylin and eosin. A blinded observer determined the cross-sectional areas of the basilar artery lumen with computerized image analysis (NIS-Elements 4.3, Nikon®, Champigny sur Marne, France).

### Statistics

The number of rats per group was assessed in a power analysis performed with online software (https://www.dssresearch.com/resources/calculators/sample-size-calculator-percentage/). We expected terutroban to increase the ^99m^Tc-HMPAO uptake by 50% compared to the SAH group. Past studies showed that cerebral blood flow decreased after a double intracisternal injection in the rat model of SAH [26]. Based on these data, we estimated that a minimum of 14 animals for both samples would be required, for an empiric *α* error level of *P* < 0.05 and a *β* error level = 0.4. Therefore, we included rats to reach a minimal sample of 14 rats in each radiolabeled study group, taking into account mortality and radiolabeled injection failure. All data are presented as the mean and standard deviation (SD). Between-group comparisons was conducted with a one- or two-way ANOVA (depending on the outcome) followed by Tukey’s multiple comparisons test. Adjusted *P* values < 0.05 were considered significant. Statistical analyses were performed with Prism® 6.0 (GraphPad Software, Inc., La Jolla, CA). The datasets generated and/or analysed during the current study are available in the “figshare” repository, with the identifier https://figshare.com/s/f7f8f15e1d185ad7e7f4.

## Results

Of the 78 rats included, 58 were exposed to intracisternal injections. Among them, 45 rats completed the nanoSPECT imaging protocol. Mortality (22.4%) occurred during the intracisternal injections. Three rats were excluded due to radiolabelled agent extravasation during injection (Fig. 1).

### SAH-induced synthesis of TP receptor agonists

**Fig. 2:**
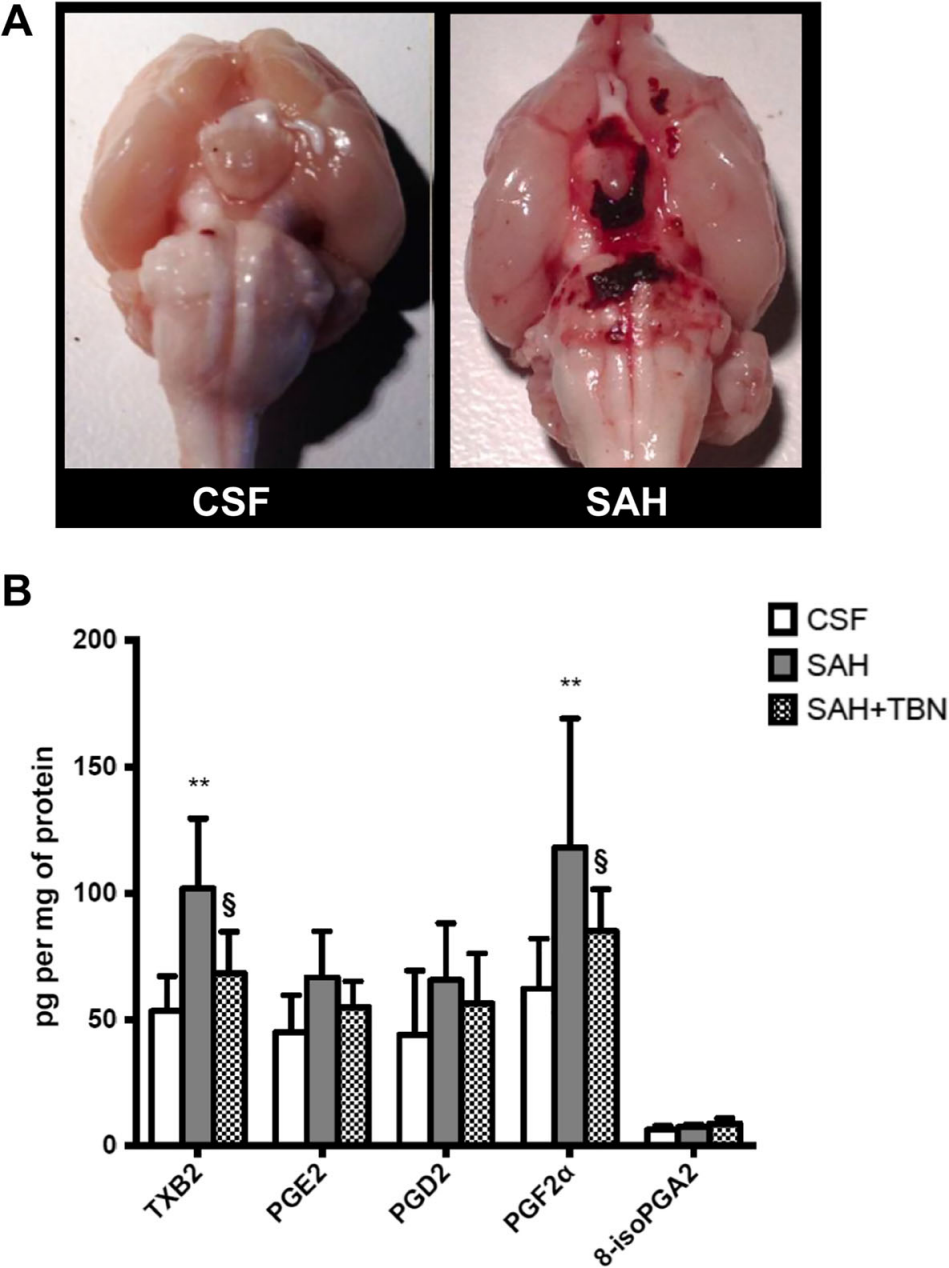
SAH-induced synthesis of TP receptor agonists. **a** Dissected brains show subarachnoid blood clots preferentially concentrated in the basal cisterns and around the brainstem in the SAH group. No blood was found in the CSF group. **b** Quantification of cerebral TP receptor agonists on day 5. Bars indicate the mean ± SD measured in rats of CSF group (*n* = 6), SAH group (*n* = 7), or SAH+TBN group (*n* = 5). **P < 0.05 compared to CSF, §*P* < 0.05 compared to SAH. CSF, cerebrospinal fluid; SAH, subarachnoid haemorrhage; TBN, terutroban; CH, cerebral hemispheres; CB, cerebellum; BS, brainstem

The model of SAH was validated by the presence of blood clots in harvested brain of all rats in the SAH and SAH+TBN groups but not in the CSF group (Fig. 2a). Post mortem quantification of cerebral TP receptor agonist revealed the following: in the SAH group, cerebral TXB-2 and PGF2α were significantly increased compared to the CSF group (101 ± 28 versus 53 ± 14 pg per mg of protein; *P* < 0.001 and 126 ± 51 versus 62 ± 20 pg per mg of protein; *P* < 0.001 respectively). Cerebral TXB-2 and PGF-2α levels were significantly reduced in the SAH+TBN group compared to the SAH group (TXB-2 68 ± 16 versus 101 ± 28 pg per mg of protein; *P* = 0.02; PGF-2α 85 ± 16 versus 118 ± 50; *P* = 0.03; Fig. 2b).

### Functional outcome

**Fig. 3:**
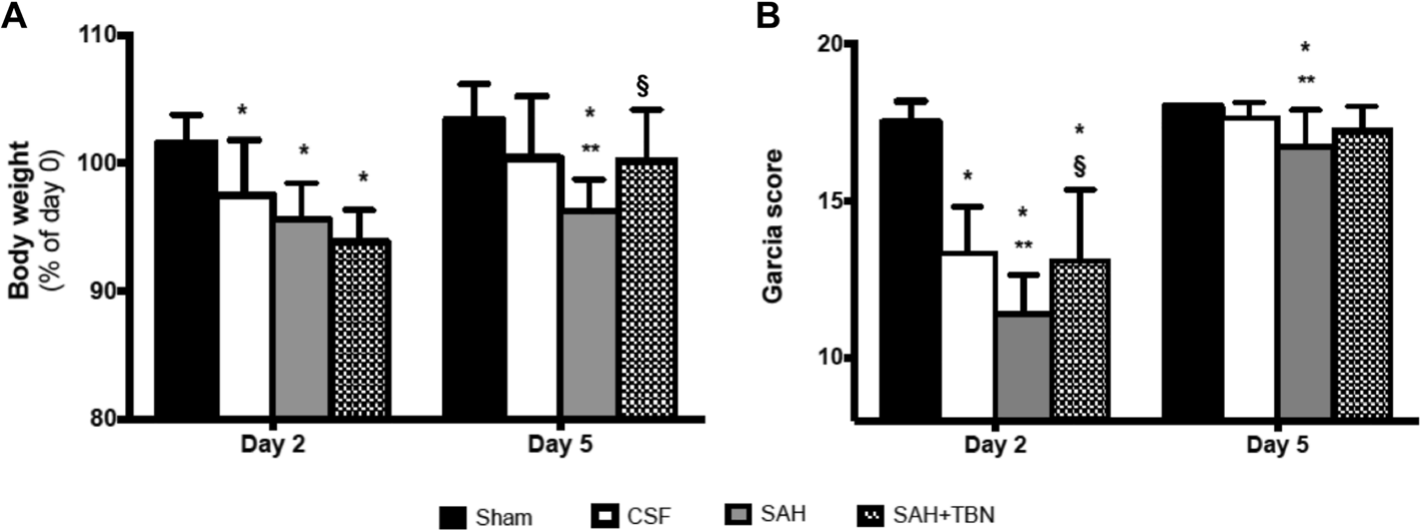
Early (day 2) and delayed (day 5) functional outcomes. **a** Changes in body weights (% of day 0). Bars indicate the mean and SD measured in rats of sham group (*n* = 13), CSF group (*n* = 15), SAH group (*n* = 15) or SAH+TBN group (*n* = 14). **b** Garcia scores measured on days 2 (D2, early effects) and 5 (D5, delayed effects). Bars indicate the mean and SD measured in rats of sham group (*n* = 12), CSF group (*n* = 15), SAH group (*n* = 15) or SAH+TBN group (*n* = 12); *P < 0.05 compared to sham, **P < 0.05 compared to CSF, §*P* < 0.05 compared to SAH. CSF denotes cerebrospinal fluid; SAH, subarachnoid haemorrhage; TBN, terutroban

On day 5, SAH rats treated with TBN (SAH+TBN group) displayed significant less weight loss than the non-treated SAH rats (SAH group) (Fig. 3a) showing a beneficial effect of TBN. This difference was not yet visible on day 2. TBN also improved neurological deficits as measured using the Garcia scale. On day 2 the SAH+TBN group displayed a significant higher Garcia score than the SAH group. On day 5, differences in the Garcia score remain significant between the SAH and the sham or CSF groups (Fig. 3b).

### ^99m^Tc-DTPA nanoSPECT imaging

**Fig. 4:**
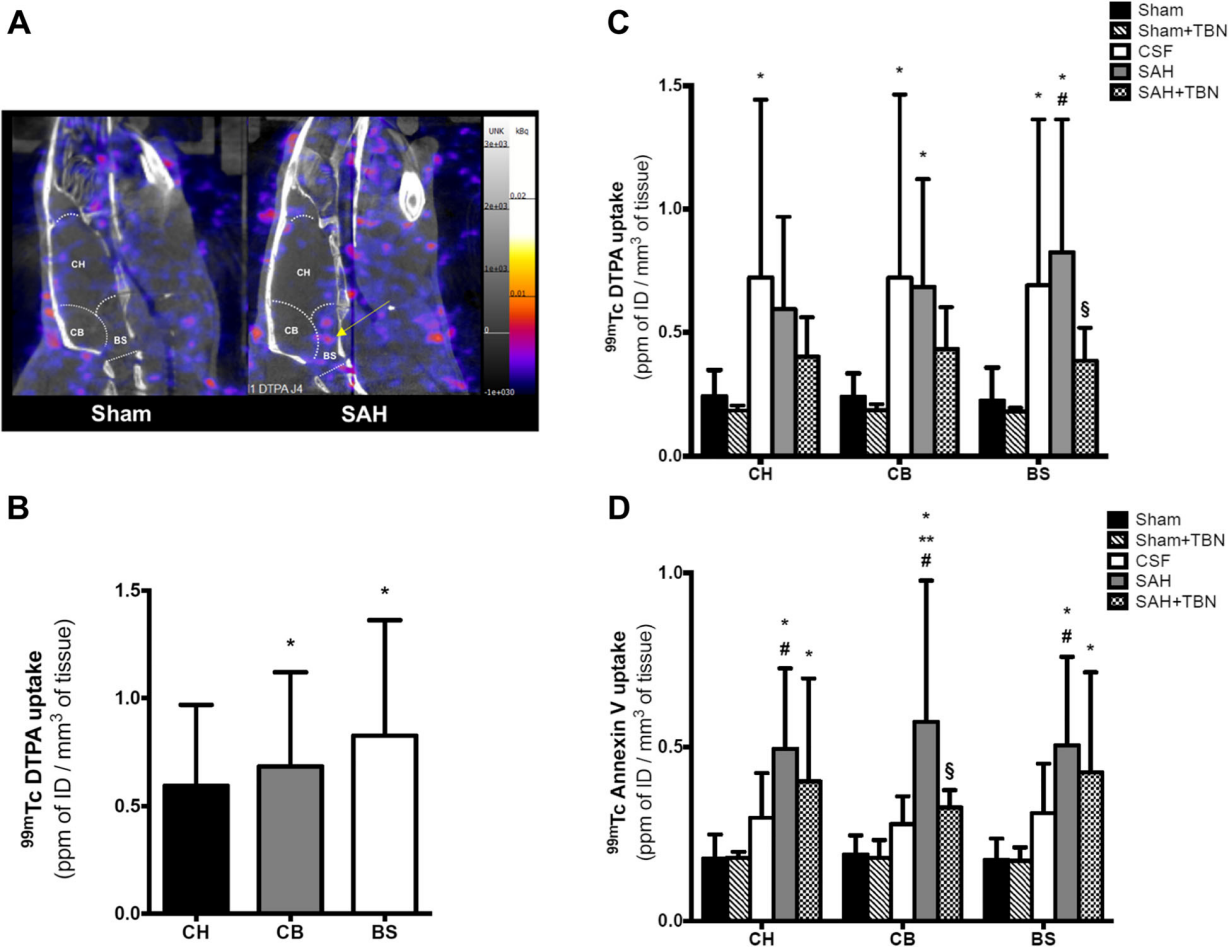
NanoSPECT study of blood-brain barrier permeability (^99m^Tc-DTPA) on day 3 and apoptosis (^99m^Tc-Anx-V128) on day 4. **a** Representative image of the differential brainstem ^99m^Tc-DTPA uptake in the SAH group (yellow arrow). Colour scales indicate signal intensities from low (blue) to high (white). NanoSPECT images (sagittal view) show the three ROIs (CH, cerebral hemispheres; CB, cerebellum; BS, brainstem). Bars indicate the mean ± SD measured in rats of sham group (*n* = 15), sham+TBN group (*n* = 5), CSF group (*n* = 14), SAH group (*n* = 15) or SAH+TBN group (*n* = 15). **b** Inter-ROI analysis of ^99m^Tc DTPA uptake in the SAH group: predominating uptake in the infra-tentorial ROIs (CB and BS). *P < 0.05 compared to CH. **c** ^99m^Tc-DTPA and **d** ^99m^Tc-Anx-V128 nanoSPECT study. Results were expressed as parts per million of injected dose (ppm ID)/mm^3^ of tissue. Bars indicate the mean ± SD measured in rats of sham group (*n* = 14), sham+TBN group (*n* = 5), CSF group (*n* = 14), SAH group (*n* = 14) or SAH+TBN group (*n* = 15). *P < 0.05 compared to sham, **P < 0.05 compared to CSF, #*P* < 0.05 compared to sham+TBN, §*P* < 0.05 compared to SAH. CSF denotes cerebrospinal fluid; SAH, subarachnoid haemorrhage; TBN, terutroban; CH, cerebral hemispheres; CB, cerebellum; BS, brainstem

On day 3, ^99m^Tc-DTPA uptake was significantly increased in the SAH group compared to the sham group in the brainstem (0.8 ± 0.5 ppmID/mm^3^ versus 0.2 ± 0.1 ppmID/mm^3^, *P* < 0.001; Fig. 4a, c) and in the cerebellum (0.7 ± 0.4 ppmID/mm^3^ versus 0.2 ± 0.1 ppmID/mm^3^, *P* = 0.03). In the SAH group, an inter-ROI analysis showed that brainstem and cerebellar uptakes were significantly higher than the cerebral hemispheres uptake (*P* = 0.02 versus cerebellum; *P* = 0.01 versus brainstem; Fig. 4a, b). No difference was found between CSF and SAH groups in all studied ROIs. TBN had no significant effect on ^99m^Tc-DTPA uptakes of sham-operated animals. In the SAH+TBN group, a significant reduction in brainstem ^99m^Tc-DTPA uptake was found compared to the SAH group (0.4 ± 0.1 ppmID/mm^3^ versus 0.8 ± 0.5 ppmID/mm^3^; *P* = 0.03; Fig. 4c), but no significant effect was found in the cerebellum (SAH+TBN versus SAH; *P* = 0.46) and the cerebral hemispheres (SAH+TBN versus SAH; *P* = 0.7).

### ^99m^Tc-Annexin V-128 nanoSPECT imaging

On day 4, ^99m^Tc-Anx-V128 uptake was significantly increased in the SAH group compared to the sham group in the brainstem (0.5 ± 0.2 ppmID/mm^3^ versus 0.2 ± 0.1 ppmID/mm^3^, *P* < 0.001), in the cerebellum (0.6 ± 0.4 ppmID/mm^3^ versus 0.2 ± 0.1 ppmID/mm^3^, *P* < 0.001) and in the cerebral hemispheres (0.5 ± 0.2 ppmID/mm^3^ versus 0.2 ± 0.1 ppmID/mm^3^, *P* < 0.001; Fig. 4d). Compared to the CSF group, ^99m^Tc-Anx-V128 uptake was significantly increased in the cerebellum (0.6 ± 0.4 ppmID/mm^3^ versus 0.3 ± 0.1 ppmID/mm^3^, *P* = 0.001). TBN had no significant effect on ^99m^Tc-Anx-V128 uptakes of sham-operated animals. TBN significantly reduced cerebellar ^99m^Tc-Anx-V128 uptake induced by SAH (0.3 ± 0.1 ppm ID/mm^3^ versus 0.6 ± 0.4 ppmID/mm^3^; *P* = 0.01; Fig. 4d). However, TBN did not show any significant effect on SAH-induced ^99m^Tc-Anx-V128 uptake in the brainstem (SAH+TBN versus SAH; *P* = 0.83) and in the cerebral hemispheres (SAH+TBN versus SAH; *P* = 0.71).

### ^99m^Tc-HMPAO nanoSPECT imaging

**Fig. 5:**
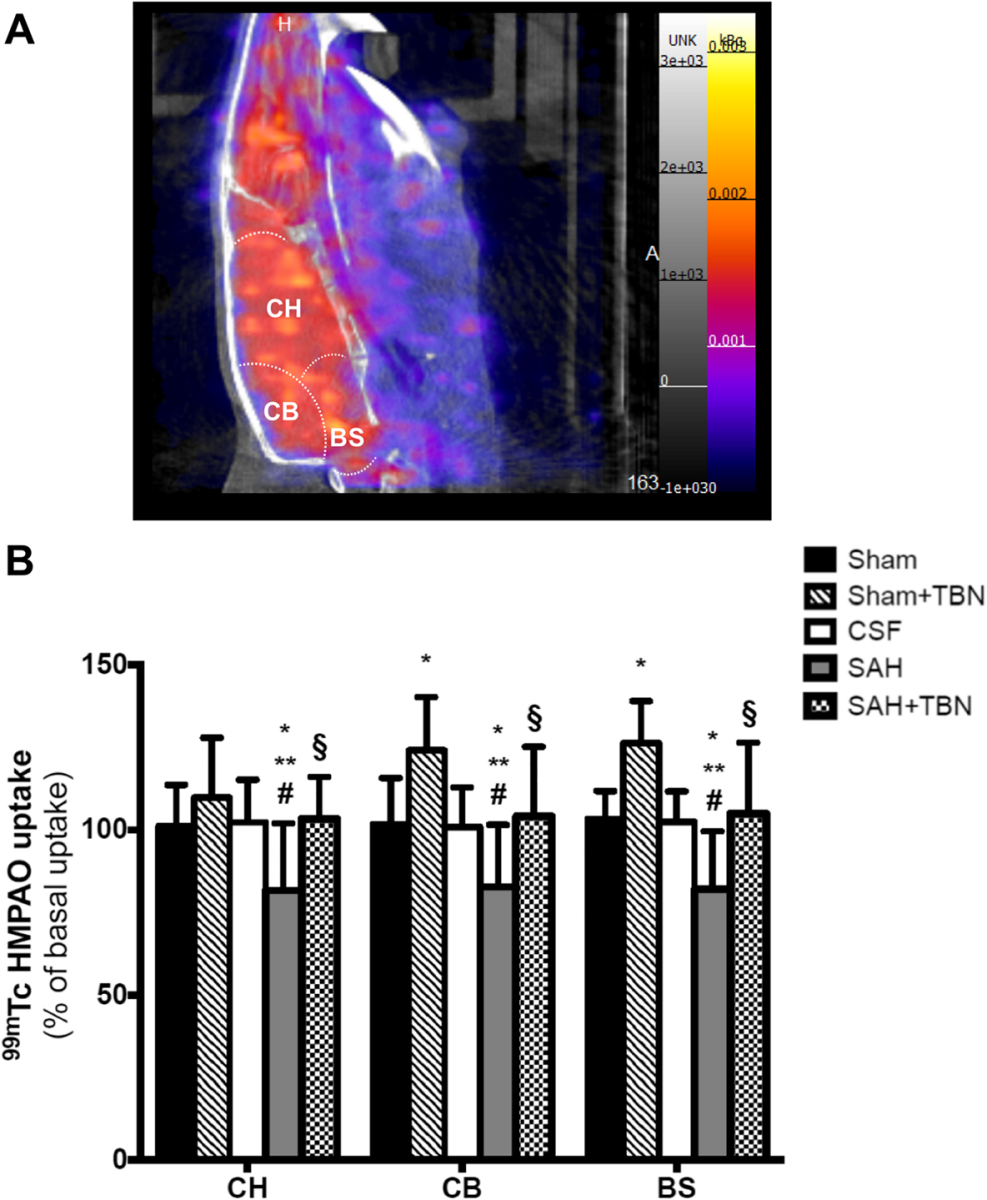
NanoSPECT study of cerebral perfusion (^99m^Tc-HMPAO) on day 5. **a** ^99m^Tc-HMPAO nanoSPECT imaging (sagittal view) shows an example of the ^99m^Tc-HMPAO brain uptake at day 5. Colour scales indicate signal intensities from low (blue) to high (yellow or white). **b** ^99m^Tc-HMPAO uptake (% basal uptake) was measured on day 5. Bars indicate the mean ± SD measured in rats of sham group (*n* = 15), sham+TBN group (*n* = 5), CSF group (*n* = 15), SAH group (*n* = 15) or SAH+TBN group (*n* = 15). *P < 0.05 compared to sham, **P < 0.05 compared to CSF, #*P* < 0.05 compared to sham+TBN, §*P* < 0.05 compared to SAH. CSF denotes cerebrospinal fluid; SAH, subarachnoid haemorrhage; TBN, terutroban; CH, cerebral hemispheres; CB, cerebellum; BS, brainstem

On day 5, ^99m^Tc-HMPAO uptakes (Figs. 5a, b) were significantly reduced in the SAH group compared to the CSF group in all studied ROIs: cerebral hemispheres (− 18 ± 20 versus 2 ± 13% of basal uptakes; *P* = 0.003), cerebellum (− 17 ± 18 versus 1 ± 12% of basal uptakes; *P* = 0.01) and brainstem (− 18 ± 17 versus 2 ± 9% of basal uptakes; *P* = 0.004). In the sham+TBN group, we found increased day 5 ^99m^Tc-HMPAO uptakes: + 9 ± 20% of basal uptakes in the cerebral hemispheres (*P* = 0.8 versus sham group), + 24 ± 16% of basal uptakes in the cerebellum (*P* = 0.05 versus sham group), and + 26 ± 12% of basal uptakes in the brainstem (*P* = 0.04 versus sham group). TBN also significantly prevented the SAH-induced reduction in ^99m^Tc-HMPAO uptake in all examined ROIs: cerebral hemispheres (3 ± 12% of basal uptakes; *P* = 0.002 versus SAH group), cerebellum (4 ± 21% of basal uptakes; *P* = 0.002 versus SAH group) and brainstem (5 ± 21% of basal uptakes; *P* < 0.001 versus SAH group; Fig. 5b).

### Evaluation of the basilar artery lumen

**Fig. 6:**
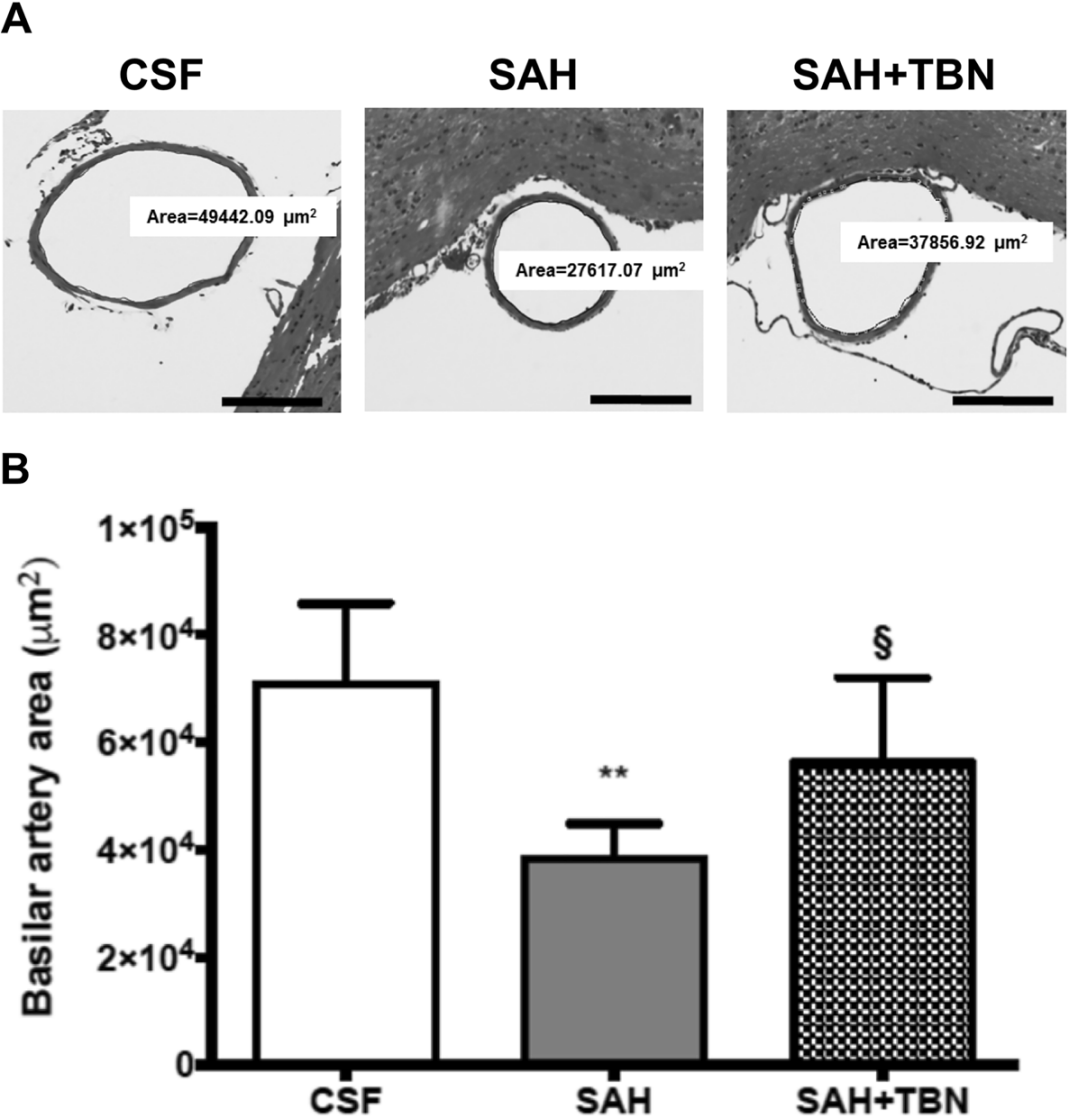
Basilar artery measurements. **a** Representative photomicrographs showing significant vasoconstriction of basilar arteries on day 5 after subarachnoid haemorrhage induction. Vasoconstriction was attenuated with terutroban (SAH+TBN). Tissue samples were stained with haematoxylin and eosin; × 200 magnification; scale bar = 200 μm. **b** Basilar artery luminal areas (μm^2^). Bars indicate the mean and SD measured in rats of CSF group (*n* = 6), SAH group (*n* = 7), or SAH+TBN group (*n* = 6). **P < 0.05 compared to CSF, §*P* < 0.05 compared to SAH. CSF denotes cerebrospinal fluid; SAH, subarachnoid haemorrhage; TBN, terutroban

Histological evaluation of the basilar artery on day 5 showed that its lumen was significantly narrowed in the SAH group compared to the CSF group (38,382 ± 6528 μm^2^ versus 70,933 ± 14,994 μm^2^; *P* < 0.001). Terutroban significantly diminished basilar artery narrowing (56,335 ± 15,728 μm^2^) compared to the SAH group (*P* = 0.04; Fig. 6a, b).

## Discussion

In the current study, we evaluated the neuroprotective effects of the TP receptor antagonist: TBN, in an experimental SAH rat model. The major findings were as follows: prevented disruption of the blood-brain barrier in the brainstem, alleviated apoptosis in the cerebellum and counteracted global delayed cerebral hypoperfusion. To our knowledge, this study is also the first to describe a rodent SAH model with nanoSPECT-CT imaging.

The blood-brain barrier disruption process has been well described after SAH [33]. Interestingly, we found that ^99m^Tc-DTPA uptakes increased in the CSF and SAH groups, particularly in the infra-tentorial ROIs (brainstem and cerebellum), near the site of injection. With regard to these results, we hypothesize that volume expansion and subsequent global cerebral ischemia might contribute to the blood-brain barrier rupture as a part of the early brain injury. ^99m^Tc-Anx-V128 has been developed for the in vivo quantification of apoptotic activities. We have already correlated ^99m^Tc-Anx-V128 nanoSPECT imaging with histological TUNEL staining during focal cerebral ischemia in rats [34]. In this work, we have found a large and global increase of ^99m^Tc-Anx-V128 uptakes in the SAH group indicating an activation of apoptosis. SAH-related apoptosis in both neuronal and endothelial cell is recognized as a major mechanism of the brain injury in the first steps of the disease [6]. Finally, ^99m^Tc-HMPAO is well-validated for exploring cerebral perfusion [31]. A significant decrease in HMPAO uptake was found on day 5 in the SAH group, but not in the CSF group. This highlighted the notion that injection of blood in the subarachnoid space may have contributed to cerebral hypoperfusion [2].

The quantification of cerebral TP receptor agonists revealed a dramatic increase of two well-described inflammation-induced vasoconstrictors after the intracisternal blood injection: TXB-2 [35] and PGF2α [36, 37]. This result validates our model regarding the induction of a cerebral inflammatory response. Thromboxane A2, as well as other prostaglandins, has already been targeted by different anti-inflammatory and antiplatelet agents in previous SAH studies [38]. The use of a TXA2-synthetase inhibitor has improved cerebral blood flow and prevented vasospasm in two experimental studies [39, 40]. Two clinical studies [41, 42] have explored the efficacy of OKY-36046 (Cataclot®), a TXA-2 inhibitor. Despite small sample sizes, they both showed a protective effect on cerebral vasospasm. By contrast, TBN pharmacological properties have already been well described in humans and the drug has been safely evaluated in a large clinical trial [23]. By antagonizing the TP receptor, it jointly inhibits the activity of enzymatic (eicosanoids) and non-enzymatic arachidonic acid metabolites (isoprostanes, 20-hydroxyeicosatetraeonic acid). In our work, TBN also diminished brain TXB-2 and PGF-2α syntheses. A similar result has been found in the serum of apo E-deficient mice treated by TBN [43], but the mechanism of this effect on agonist synthesis is unknown. As agonists are synthetized by cells expressing TP receptors on their surface (endothelial cells and platelets), a better understanding of TP receptor intracellular signalling could be of interest.

Our results suggest that TBN exerts protection on the brainstem blood-brain barrier. The protective effect of TBN may be secondary to its endothelioprotective effect, as it has been previously described in the animal with reduced endothelial ICAM-1 expression and leukocyte recruitment [43–45]. Indeed, endothelial inflammation, expression of adhesion molecule and leukocyte recruitment are well-known mechanisms of blood-brain barrier disruption [39]. Moreover, Siler et al. have found a decreased expression of VCAM-1 and less cerebral oedema in mice exposed to epoxyeicosatrienoic acids, a recognized TP receptor antagonist [46], after SAH [47]. Finally, TP receptor agonists were already found to enhance endothelial apoptosis by inhibiting cell survival properties of TNFα during inflammation and ischemia-reperfusion [48].

Our data demonstrate a general beneficial effect of TBN on brain perfusion on day 5. An increase in ^99m^Tc-HMPAO uptake was found in sham-operated and in SAH rats exposed to TBN. This action was probably secondary to an intrinsic vasodilatory property. In support of this view, ^99m^Tc-HMPAO SPECT imaging has been validated for determining cerebral perfusion effects of cerebrovasoactive drugs acting via multiple modes of pharmacological action (such as nimodipine or acetazolamide) in primates [49]. Likewise, our results suggest a beneficial effect of TBN on basilar artery vasomotricity. Finally, a microvascular dysfunction could impair cerebral perfusion after SAH [50]. Recent studies showed that TBN could promote cerebral arterioles’ vasodilation and preserve regulative properties of endothelial cells, by protecting the function of endothelial nitric oxide synthase and inhibiting the Rho-kinase pathway [51, 52].

### Limitations

The double autologous arterial blood intracisternal injection model had been recognized for its validity [26] and low mortality rate [53], but this model has its limitations due to the absence of vascular rupture. We have not found an increase in cerebral 8-isoPGA2 level after blood injection revealing the poor activation of the oxidative stress response in this animal model. Nonetheless, this minimally invasive rat model, by avoiding cutaneous incision and burr-hole drilling, reduces procedure-related intracranial artefacts that could be potentially detected by the highly sensitive nanoSPECT technique. Even if same volumes of blood or CSF were injected, after a strict confirmation of the intracisternal position, we do not have any data on intracranial pressure or cerebral blood flow measurement. Then, the Garcia score in this animal model lacks sensibility, and other functional tests would have been needed for a better neurobehavioural assessment of treatment effect [54]. We have found heterogeneous effects of TBN depending on the type of radiotracers and the considered ROI. For example, TBN protects BBB in the brainstem without exerting a significant effect on apoptosis in this ROI. This topographical discrepancy can be related to the site of blood injection with a diverse effect on each ROI and/or to a lack of power because of too small sample sizes. Despite our past work [34], our study lacks a histologic correlation of apoptosis with ^99m^Tc-Anx-V128 uptake in this specific model. ^99m^Tc-Anx-V128 uptake could also be enhanced by blood and subarachnoid clots as annexin V has an affinity for blood components such as activated platelets. This possibility may reduce ^99m^Tc-Anx-V128 specificity for apoptotic cells in SAH models. As we have found in the cerebellum ROI, TBN may play a role in apoptosis prevention. Regarding the limits of ^99m^Tc-Anx-V128, confirmation of this result is needed with other type of investigations dedicated to apoptosis quantification. Finally, additional studies are needed to better understand the relation between the different post-SAH events (hypoperfusion, BBB disruption, apoptosis…) and to specify the mechanistic aspects of TBN activity.

## Conclusions

With in vivo nanoSPECT imaging in a rodent model of SAH, this study shows that TBN administration after SAH protects the blood-brain barrier, improves cerebral perfusion and may prevent apoptosis. Thus, this TP receptor antagonist shows a promising result in treating post-SAH neurovascular events.

## Abbreviations

Anx: Annexin
BS: Brainstem
CB: Cerebellum
CH: Cerebral hemispheres
CSF: Cerebrospinal fluid
CT: Computed tomography
DTPA: Diethylenetriaminepentacetate
HMPAO: Hexamethylpropyleneamineoxime
ID: Injected dose
MBq: Megabecquerel
MS: Mass spectrometry
PG: Prostaglandin
ppm: Parts per million
ROI: Region of interest
SAH: Subarachnoid haemorrhage
SPECT: Single-photon emitted computed tomography
TBN: Terutroban
Tc: Technetium
TP: Thromboxane and prostaglandins
TXB-2: Thromboxane-B2
